# Whole-genome association studies of alcoholism with loci linked to schizophrenia susceptibility

**DOI:** 10.1186/1471-2156-6-S1-S9

**Published:** 2005-12-30

**Authors:** Junghyun Namkung, Youngchul Kim, Taesung Park

**Affiliations:** 1 Bioinformatics Program, Seoul National University, Seoul 151-747, South Korea; 2 Department of Statistics, Seoul National University, Seoul 151-747, South Korea

## Abstract

**Background:**

Alcoholism is a complex disease. There have been many reports on significant comorbidity between alcoholism and schizophrenia. For the genetic study of complex diseases, association analysis has been recommended because of its higher power than that of the linkage analysis for detecting genes with modest effects on disease.

**Results:**

To identify alcoholism susceptibility loci, we performed genome-wide single-nucleotide polymorphisms (SNP) association tests, which yielded 489 significant SNPs at the 1% significance level. The association tests showed that tsc0593964 (*P*-value 0.000013) on chromosome 7 was most significantly associated with alcoholism. From 489 SNPs, 74 genes were identified. Among these genes, *GABRA1 *is a member of the same gene family with *GABRA2 *that was recently reported as alcoholism susceptibility gene.

**Conclusion:**

By comparing 74 genes to the published results of various linkage studies of schizophrenia, we identified 13 alcoholism associated genes that were located in the regions reported to be linked to schizophrenia. These 13 identified genes can be important candidate genes to study the genetic mechanism of co-occurrence of both diseases.

## Background

Alcoholism is a complex disease that tends to run in families. It has been reported that alcoholism is accompanied by many other psychiatric disorders, including schizophrenia [1]. The co-occurrence of these psychiatric disorders and alcoholism can be explained in several ways. First, patients may use alcohol to relieve the symptom of mental diseases. Second, disruption of neurochemical systems may cause psychiatric disorders and alcoholism simultaneously. Several molecules including serotonin, neuropeptide Y, and dopamine have been studied to explore this hypothesis. Third, the genes responsible for these diseases may transmit together because they are closely linked on a chromosome and co-segregate without any functional relationship.

Linkage or association study of polymorphisms may provide useful information on the genetic mechanism of co-occurrence of psychiatric disorders and alcoholism. Among psychiatric disorders, schizophrenia is also known to be affected by multiple genes [2]. Schizophrenia patients with substance abuse problems, especially alcoholics, are clinically important because they usually have a poor prognosis [3,4].

In this study, we are interested in determining whether there is any common genetic factor that may increase the susceptibility of alcoholism and schizophrenia simultaneously. Although linkage analysis has been a successful choice for genetic analysis of Mendelian diseases, association analysis has shown to have greater power than linkage analysis when used to detect genes with modest effect on disease [5]. Thus, we conduct genome-wide association tests to find alcoholism susceptibility loci by analyzing the Collaborative Study on the Genetics of Alcoholism (COGA) data from the Genetic Analysis Workshop 14 (GAW 14). Because COGA data were collected from multiplex families, we consider only the approaches that take advantage of the whole pedigree data.

In our analysis, we primarily use the pedigree disequilibrium test (PDT) [6]. The PDT is an extension of the transmission disequilibrium test (TDT) [7], and it has been widely applied to the analysis of large pedigree data including multiple nuclear family or sibling pairs. We test association for both alleles and genotypes using the PDT. Additionally, we apply the generalized estimating equations (GEE) approach [8] to test the association for each genotype with the disease while adjusting for some phenotypic covariates. By using both the PDT and GEE approaches we obtain candidate markers for alcoholism susceptibility and then compare them with the loci that are reported to be linked to schizophrenia.

## Methods

Disease affection status was defined by using both ALDX1 (DSM-III-R+Feighner) and ALDX2 (DSM-IV). A sample with ALDX1 = 5 or ALDX2 = 5 was defined as the affected, and ALDX1 = 1 or ALDX2 = 1 as the unaffected. Others treated as non-informative data. With these criteria, 668 were classified as "affected" and 285 as "unaffected" from 1,614 individuals.

### Linkage analysis

For the comparison of association with the linkage analysis, we first performed nonparametric linkage (NPL) analysis for microsatellites. Due to computational problems, NPL analysis for single-nucleotide polymorphisms (SNPs) was not performed. The linkage analysis for microsatellites was performed by using NPL procedure of SIMWALK2 software [9]. To transform GAW14 data into input format for SIMWALK2 software, we used MEGA2 software [10].

### PDT

To test for association of microsatellites and SNPs with disease, we used the PDT. Because the PDT is a family-based association test, it can avoid problems of false positives caused by population stratification. There are two types of the PDT available: allele-based PDT and genotype-based PDT (genotype-PDT). For the allele-based PDT, two test statistics are commonly used: sum-PDT and average-PDT. The sum-PDT gives more weight to families with a larger number of multiple affected individuals. In contrast, the average-PDT gives equal weight to all families [11]. The genotype-PDT tests for association between genotypes and disease. This is more powerful than the allele-based PDT when the genetic effect of an allele is dominant or recessive rather than additive. The genotype-PDT can test multilocus effects without the ambiguities associated with haplotype analysis, and allow for testing interactions among markers [12]. We used the genotype-PDT to test for association between disease and genotypes at a single locus as well as at multiple loci. All the tests were conducted by using PDT 5.1 software.

### GEE

The GEE approach accounts for familial correlation in the analysis and allows for adjustment for covariates such as sex and age in selecting significant genotypes of SNPs. For the binary response of disease affection status, the model is given by

Logit [pr(*D*_*ij*_)] = *β*_0 _+ *β*_age_age_*ij *_+ *β*_sex_sex_*ij *_+ *β*_geno_genotype_*ij*_, where

*D*_*ij *_is the affection status of *j*^th ^individual in *i*^th ^family

(affected = 1, unaffected = 0)

We conducted this analysis using R software .

### Gene finding

After identifying SNPs associated with alcoholism, we obtained gene information from the dbSNP website . The additional information of genes such as chromosomal location and functional annotation was obtained from the SOURCE website . Then, we compared the genes and regions in which they are located to published results of various linkage studies of schizophrenia.

## Results

### Preliminary analysis using microsatellites

Before performing the association analysis using large number of SNPs, we conducted linkage and association analyses for microsatellite markers. At first, a chromosome-wide association test was performed using two PDT statistics: average-PDT and sum-PDT. At the 5% significance level (α = 0.05) for either the sum-PDT or average-PDT, this preliminary test shows that 6 microsatellites from chromosomes 6 and 8 tend to have associations with alcoholism. Among them, two microsatellites are located adjacent to the schizophrenia susceptibility region [2,13]: 1) D6S474 (6q21) with *p*-values 0.0182 for the sum-PDT and 0.0144 for the average-PDT, 2) D8S1106 (8p21 SCZD6) with *p*-value 0.0155 for the sum-PDT.

To test for linkage of markers to alcoholism, we performed NPL analysis. Chromosome 7 showed two peaks of moderate linkage disequilibrium with alcoholism at D7S673 (30.1 cM) with -log *p*-value 2.077 (NPL-PAIR) and D7S820 (107.5 cM) with 2.041.

### Association test for SNPs

From 15,878 autosomal SNPs, we first selected SNPs that have *p*-values smaller than 0.05 for both allele based PDTs. One hundred and ninety SNPs had *p*-values less than 0.01 and 18 SNPs had *p*-values less than 0.001 by either of the two PDTs. Using the genotype-PDT we identified 138 and 16 SNPs showing significant association at α = 0.01 and 0.001, respectively. Among 138 SNPs, 94 also showed significant associations for the allele-based PDT at α = 0.01.

**Figure 1:**
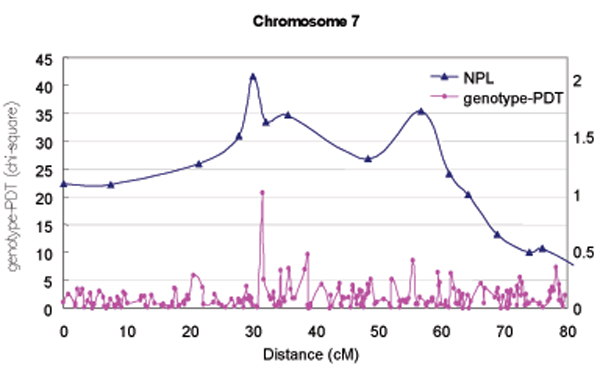
Linkage for microsatellites and association for SNPs on chromosome 7.

SNP tsc0593964 on chromosome 7 showed the most significant association with alcoholism (*p*-value = 0.000013, genotype-PDT). It is located about 2.3 cM away from the modest NPL peak of D7S673 found in the linkage analysis of microsatellites (Figure 1).

As an alternative, we also applied the GEE approach, which considers familial correlation. From this analysis, only 65 SNPs showed *p*-values less than 0.01. rs1262129 appeared to be most significant (*p*-value = 0.000068). Among the 65 significant SNPs, two SNPs were also significant for the genotype-PDT: tsc0253130 (*p*-value = 0.0007) and tsc0834636 (*p*-value = 0.0054). The number of significant SNPs is much smaller than that of PDT. It is probably due to the lack of power caused by ignoring genetic inheritance information in the GEE approach.

In addition, we performed multipoint association analysis using the multipoint genotype-PDT. The multipoint genotype-PDT tests for association of multiple loci without haplotyping. We conducted 2-point, 3-point, and 5-point analysis for the adjacent markers. We obtained 98 SNP pairs from the 2-point analysis, and 42 SNP triple sets from the 3-point analysis at the 1% significance level. The 5-point analysis resulted in no significant marker sets. Some identified SNP sets did not contain any SNP that showed significant association from the single-locus test. This underscores the importance of interactions among multiple markers to search for candidate disease genes.

Using the dbSNP and the SOURCE database website, we selected intragenic SNPs among those associated with alcoholism. A total of 74 known genes were found to contain the significant SNPs after hypothetical genes or open reading frames were excluded. Two of these genes, *GABRA1 *(tsc0325674, *p*-value = 0.0035 by the average-PDT) and *CHRNA3 *(rs1878399, *p*-value = 0.0083 by the average-PDT) are neurotransmitter receptors; *NTRK2 *(tsc0656804, *p*-value = 0.0041 by the average-PDT) has function of neurogenesis. Of note, *GABRA1 *is a member of the same gene family as *GABRA2*, which was reported recently to be associated with alcoholism [14].

Finally, we compared the chromosomal location of the 74 genes with the schizophrenia susceptibility regions [2,13]. The regions were previously identified by several linkage studies of schizophrenia. The comparison revealed that 13 genes were located in schizophrenia susceptibility region (Table 1). These genes and SNPs on them can be putative markers responsible for both alcoholism and schizophrenia susceptibility.

## Summary and Discussion

Through the analysis using microsatellites, we obtained rough regions showing modest linkage or association with alcoholism. Later, we found that the most significant SNP, tsc0593964, was located near the modest linkage peak on chromosome 7.

We conducted the genome-wide association analysis for large number of SNPs by using different association methods such as the allele-based PDT, genotype-based PDT for single locus or for multiple loci, and GEE. Each method resulted in a different number of significant markers. Thus, the results should be interpreted carefully. We think it would be important to compare the results in a more systematic way by using simulation studies in the future.

With additional biological information such as gene names and functions, the list of selected candidate markers may be quite useful for the study of complex disease, even though the 1% significance level is not stringent enough for the genome-wide tests. We searched for gene information of the selected SNPs and found that some of the genes are related to the neurochemical system.

The evidence for significant co-morbidity of substance abuse, especially alcoholism, and schizophrenia is very robust [4]. The genetic relationship between two diseases has not yet been explained. We think our findings of the 13 genes, which are associated with alcoholism and also located in schizophrenia linkage regions, can be helpful to study genetic factors responsible for both diseases.

## Abbreviations

COGA: Collaborative Study on the Genetics of Alcoholism

GAW14: Genetic Analysis Workshop 14

GEE: Generalized estimating equation

NPL: Nonparametric linkage

PDT: Pedigree disequilibrium test

SNP: Single-nucleotide polymorphism

TDT: Transmission disequilibrium test

## Figures and Tables

**Table 1:** The SNPs showing significant association located in major schizophrenia susceptibility candidate regions

Chr	SNP ID	Gene symbol	Cytoband	PDT *p*-value	Reported schizophrenia region^e^
1	rs908857	DUSP10	1q41	0.0043^a^	1q32.2-q41
	rs1053074-rs10594	KCNJ10	1q22-q23	0.0055^c^	1q21-22
	tsc1457991-tsc1254625	PBX1	1q23	0.001^c^	1q22-23
					
5	rs1229708	SPOCK	5q31	0.009^a^	5q31
	tsc0935735	FER	5q21	0.0088^b^	5q21-31
					
6	rs1498426	CDKAL1	6p22.3	0.006^b^	6p22-24
	rs1022092	SLC16A10	6q21-q22	0.0039^b^	
	tsc0046065	PREP	6q22	0.0022^a^	6q21-q22.3
	tsc0615608	AIM1	6q21	0.0087^c^	
	tsc0253130	TCBA1	6q21	0.0003^b^ (0.009)^d^	
					
8	tsc0149489-tsc0514918-tsc0529734	PTK2B	8p21.1	0.0056^c^	8p21-22
	rs898249-rs900267-rs310319	BIN3	8p21.3	0.0053^c^	
					
10	rs729245	CACNB2	10p12	0.0009^a^	10p11-15

^a^Average-PDT *p*-value

^b^Sum-PDT *p*-value

^c^Genotype-PDT *p*-value

^d^GEE *p*-value

^e^Data were brought from Michael et al.'s review article [2]. SNPs listed are located in genetic regions and showed allelic or genotypic association at 1% significance level. The schizophrenia susceptibility regions were identified by several independent linkage studies.
